# Effects of the alkylamine functionalization of graphene oxide on the properties of polystyrene nanocomposites

**DOI:** 10.1186/1556-276X-9-265

**Published:** 2014-05-28

**Authors:** Jinhee Jang, Viet Hung Pham, Balasubramaniyan Rajagopalan, Seung Hyun Hur, Jin Suk Chung

**Affiliations:** 1 School of Chemical Engineering, University of Ulsan, Namgu, 93 Daehakro, Ulsan, 680-749, Republic of Korea

**Keywords:** Graphene oxide, Functionalization, Alkylamine, Polystyrene, Nanocomposites

## Abstract

Alkylamine-functionalized graphene oxides (FGOs) have superior dispersibility in low-polar solvents and, as a result, they interact with low-polar polymers such as polystyrene. In this work, the functionalization of graphene oxide using three types of alkylamines, octylamine (OA), dodecylamine (DDA), and hexadecylamine (HDA), was performed, and nanocomposites of polystyrene (PS) and FGOs were prepared via solution blending. Different dispersions of FGOs over PS were obtained for the three alkylamines, and the properties of the PS composites were influenced by the length of the alkylamine. A better thermal stability was observed with a longer chain length of the alkylamine. On the other hand, functionalization with the shortest chain length alkylamine resulted in the highest increase in the storage modulus (3,640 MPa, 140%) at a 10 wt.% loading of FGO.

## Background

Graphene has attracted intensive interest due to its extraordinary electrical, thermal, and mechanical properties [1,2]. Among its wide range of applications, recent studies have demonstrated that polymer nanocomposites based on graphene have resulted in dramatic improvements in the mechanical, electrical, and gas barrier properties of pristine polymers [3-6]. Homogeneous dispersion of graphene in the polymer matrix is an essential requirement to obtain the desired properties. Graphene oxide (GO) with abundant oxygen-containing groups, such as epoxy, hydroxyl, and carboxyl, can be well dispersed in a polymer matrix due to its good interaction with polymer chains [7-9]. However, the agglomeration of graphene sheets due to van der Waals forces only allows for a successful colloidal suspension within a narrow range of organic solvents. Park et al. reported that highly reduced graphene oxide was dispersed in organic solvents with a sum of solubility parameters (δ_p_ and δ_H_) in the range of 13 to 29 [10]. Recently, it was reported that alkylamine-functionalized graphene oxide (FGO) exhibited good dispersion in solvents and a strong interfacial interaction with low-polar organic solvents and polymers [11-17]. GO modified with HDA showed superior dispersion up to 7 mg/mL in organic solvents with low Hansen solubility parameters, such as xylene and toluene [18]. Thus, they could be effectively used as a nanofiller even in low-polar polymers such as polyethylene [19,20].

In this work, three alkylamines, OA, DDA, and HDA, with different alkyl chain lengths were utilized to examine the effect of alkylamine functionalization of GO on the properties of FGO/PS composites. When the FGO/PS nanocomposites were prepared by solution blending, the FGOs were homogeneously dispersed over the PS matrix even at a high concentration in chloroform.

## Methods

### Preparation of FGO and FGO/PS nanocomposites

GO was prepared by a modified Hummers method using expanded graphite (Grade 1721, Asbury Carbons, Asbury, NJ, USA) which was heated for 10 s in a microwave oven. The ratio of GO to alkylamines (CH_3_(CH_2_)_7_NH_2_, CH_3_(CH_2_)_11_NH_2_, CH_3_(CH_2_)_15_NH_2_, Sigma Aldrich, St. Louis, MO, USA) was fixed at 1.0 g of GO to 0.01 mol of alkylamine. The alkylamine solutions were prepared by dissolving 0.010 mol of OA, DDA, or HDA in 30 mL of ethanol (SK Chemicals, Gyeonggi-do, Korea). The FGOs were produced by gradually adding the alkylamine solution into the GO solution (1.0 mg/mL) followed by stirring at 60°C for 12 h. During the alkylamine functionalization, the color of the GO solution gradually changed from yellow to black. This change was accompanied by an aggregation of graphene particles due to the hydrophobicity of the alkylamine-functionalized GO, indicating the simultaneous functionalization and slight reduction of GO [14,19]. The suspensions were filtered and washed three times with methanol. The obtained products were denoted as FGO-OA, FGO-DDA, and FGO-HDA, respectively.

For solution blending of the FGOs and PS, we selected chloroform (OCI Chemical, Seoul, Korea), which is an effective media for both FGOs and PS. Based on the amount of PS (*M*_w_ approximately 192,000 g mol^−1^, Sigma Aldrich, St. Louis, MO, USA), the FGO loadings relative to PS were fixed at 0.5, 1.0, 2.0, 3.0, 5.0, and 10.0 wt.%. Solution blending was easily performed by adding 5 g of PS into the FGO in chloroform. The resulting FGO/PS solution was stirred for 2 h followed by sonication for 30 min. After that, the FGO/PS suspension was coaggregated by pouring the solution into 1.5 L of methanol (SK Chemicals, Gyeonggi-do, Korea) under vigorous stirring for 1 h. The products were filtered and washed three times with methanol and dried at 60°C for 12 h.

### Characterizations

The compositions of the FGO/PSs were analyzed using an elemental analyzer (EA; Flash 2000, Thermo Scientific, Hudson, NH, USA). Fourier transform infrared (FT-IR) spectra were analyzed using an FT-IR spectrometer (Nicolet 380, Thermo Scientific, Madison, WI, USA). The morphologies of the freshly fractured surface of the neat PS and FGO/PS composites film were observed by scanning electron microscopy (SEM; JSM-6500FE, JEOL, Tokyo, Japan). A small amount of the FGO/PS nanocomposites was dispersed in ethanol in order to obtain meticulous field emission transmission electron microscope (FETEM; JEM-2100 F, JEOL, Tokyo, Japan) images. Thermogravimetric analysis (TGA) was performed under a nitrogen atmosphere at a heating rate of 10°C/min (Q50, TA Instruments, New Castle, DE, USA). The dynamic mechanical properties of the FGO/PS composites were measured using a dynamic mechanical analyzer (DMA-Q800, TA Instruments, New Castle, DE, USA) in the single cantilever deformation mode at a frequency of 1 Hz from 0°C to 180°C at a heating rate of 3°C/min.

## Results and discussion

**Figure 1:**
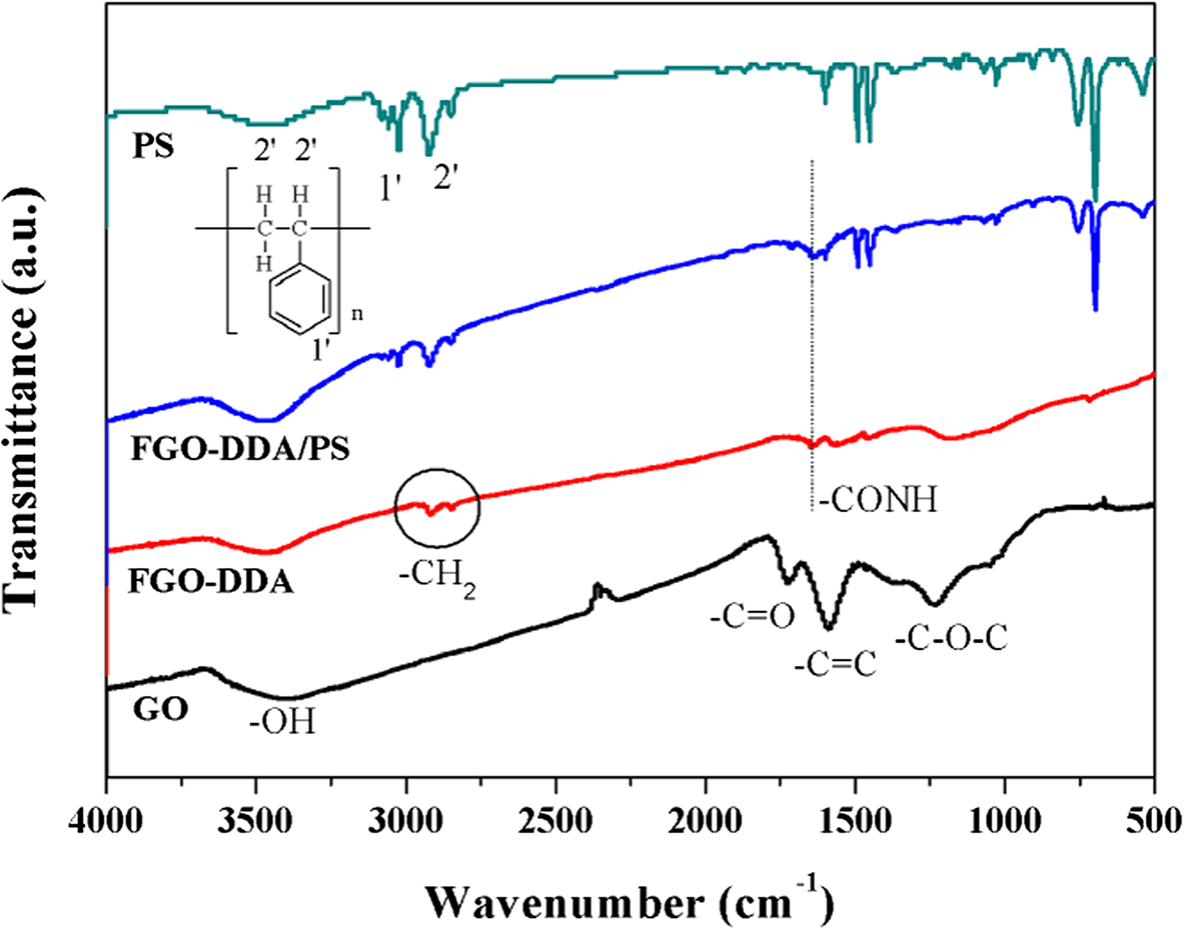
FT-IR spectra of GO, FGO-DDA, FGO-DDA/PS composites, and neat PS.

As shown in Figure 1, FT-IR was used to verify the formation of covalent bonds between GO and the alkylamines. Typical peaks for GO were obtained, including C-O-C (1,110 to 1,047 cm^−1^), C = C (1,585 cm^−1^), C = O (1,720 cm^−1^), and -OH (3,376 cm^−1^). In the case of FGO-DDA, the intensity of the C-O-C peak decreased significantly after functionalization, and two new prominent peaks appeared at 2,850 cm^−1^ and 2,920 cm^−1^, corresponding to the stretching and vibration of -CH_2_ groups, respectively, that originated from the alkylamine [21]. In addition, another new peak was observed at approximately 1,650 cm^−1^, which can be attributed to -CONH- bonding, indicating formation of a covalent bond of DDA with the GO surface, which confirms the grafting of alkylamine on FGO in the composite [13,19]. Comparing FGO-DDA/PS with pristine PS, all of the peaks from the FGO-DDA/PS composite have lower intensities, and the -CONH- peak appeared in the same region as FGO-DDA [22], which prove that FGO-DDA was associated with the PS matrix.

The elemental analysis was further used to confirm the covalent functionalization of GO with DDA. The N contents were determined to be 3.07, 3.17, 3.21, and 3.21 wt.% for reaction times of 6, 12, 18, and 24 h, respectively, while the C_graphene_/O ratios were in the range of 2.01 to 2.43. After 12 h of reaction, the C_graphene_/N ratio tended to saturate around 12.5, corresponding to one DDA molecule per six aromatic rings on the GO sheet.

**Figure 2:**
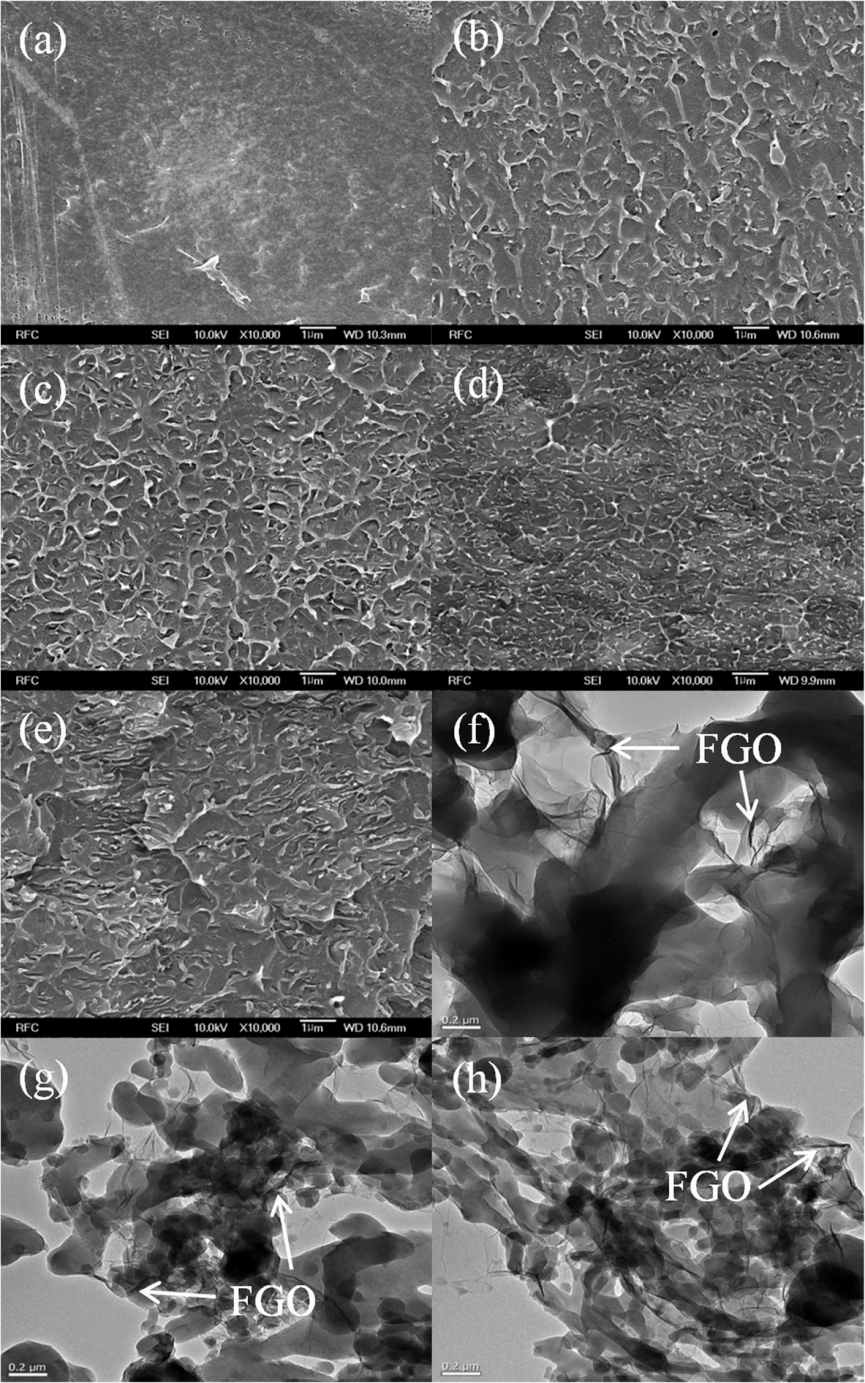
**Dispersion properties of FGO on PS.** FE-SEM images of neat PS and the FGO/PS nanocomposites: **(a)** neat PS, **(b)** 1 wt.% FGO-OA/PS, **(c)** 3 wt.% FGO-OA/PS, **(d)** 10 wt.% FGO-OA/PS, and **(e)** 10 wt.% FGO-HDA/PS. TEM images of 10 wt.% **(f)** FGO-OA/PS, **(g)** FGO-DDA/PS, and **(h)** FGO-HDA/PS.

Cross-sectional images of freshly fractured pristine PS and FGO/PS composites were observed using SEM (Figure 2a,b,c,d,e). As shown in Figure 2a,b, even with a small amount of FGO, the FGO/PS composite exhibited noticeably increased wrinkles compared to pristine PS. As the FGO content increased, the wrinkles became finer, which indicates a strong interaction between FGO and PS. It is interesting to note that all of the FGOs were homogeneously dispersed onto the PS matrix even at high loading (10 wt.%). When the chain length of the alkyl group of the FGOs was increased, the wrinkles of the FGO/PS composite became larger and wider (Figure 2d,e), which can be attributed to the effect of the increased aspect ratio of the alkylamines [23].The dispersions obtained at a 10 wt.% loading of the FGOs over PS composites were also observed by TEM (Figure 2f,g,h). Because the FGOs are compatible with the PS matrix, the FGO sheets were uniformly dispersed on the PS matrix, which is consistent with the SEM images. Notably, FGO-OA/PS showed a broad, plate-type dispersion on the transparent PS film, whereas FGO with a long length alkyl chain had a tiny droplet form on the PS film.

**Figure 3:**
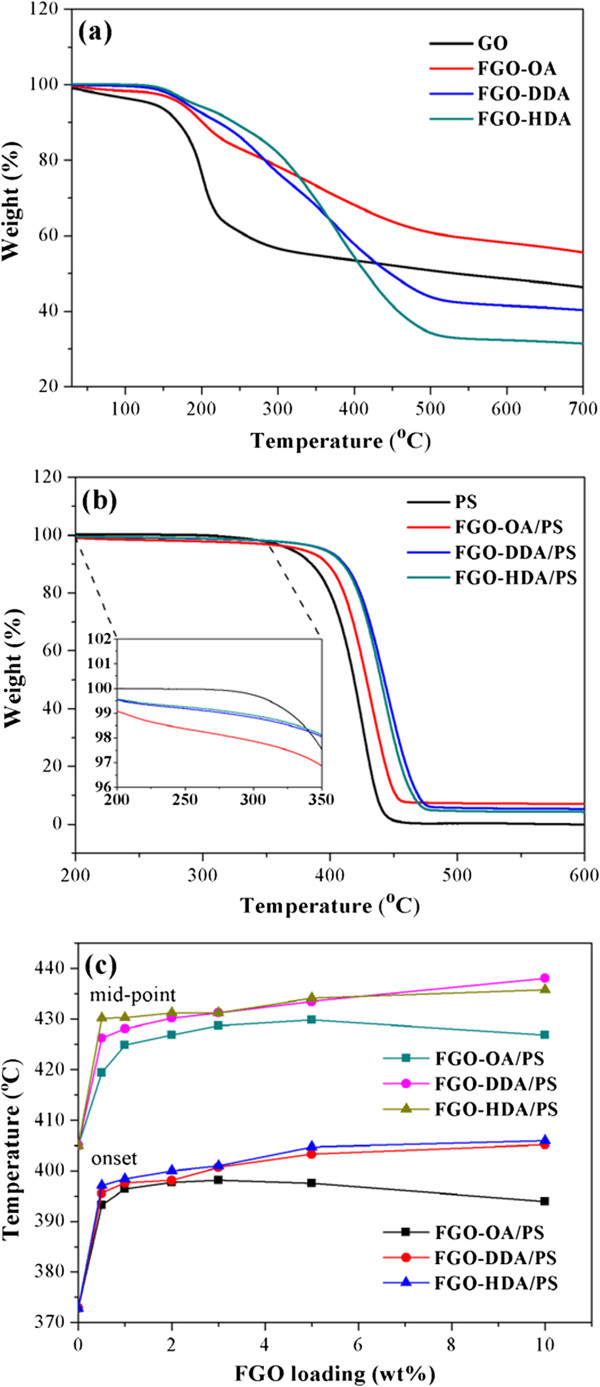
**Thermal properties of FGO/PS composites. (a)** TGA curves of GO and FGOs, **(b)** 10 wt.% FGO/PS nanocomposites, and **(c)** the onset and mid-point decomposition temperatures as a function of the FGO loading.

TGA analyses were performed to investigate the thermal properties of the FGO/PS composites and pristine PS. In the thermal stabilities of FGOs (Figure 3a), the main mass loss occurred from 200°C to 500°C due to the decomposition of the alkylamine moiety [18]. The mass residues of the FGOs decreased with increased alkylamine length, from 60 wt.% for FGO-OA to 43 wt.% for FGO-DDA and 34 wt.% for FGO-HDA at 500°C. The onset decomposition temperature (*T*_onset_) of FGO/PS composites was 20°C to 30°C higher than that of pure PS which occurred around 372°C (Figure 3b,c). FGO-HDA/PS, which has the longest alkyl chain among those tested, showed the best thermal stability. The *T*_onset_ and *T*_mid_ (mid-point of the decomposition temperature) values were 406.0°C and 435.8°C, respectively, with 10 wt.% FGO content, which are about 30°C higher than those of pristine PS. The improved thermal stability of the FGO/PS composites can be attributed to the very high aspect ratio of FGO, which is homogeneously distributed in the PS matrix, forming a tortuous path, preventing the escape of small gaseous molecules during thermal degradation [19]. However, at high loading, FGO layers with shorter alkyl chain lengths produces a less stable char layer during thermal decomposition. The lower thermal stability of FGO-OA/PS in comparison with those of FGO-DDA/PS and FGO-HDA/PS might be explained by the fact that FGO-OA has higher thermal conductivity than FGO-DDA and FGO-HDA due to short functionalized alkyl chain, which might act as heat source domain more effectively [24,25].

**Figure 4:**
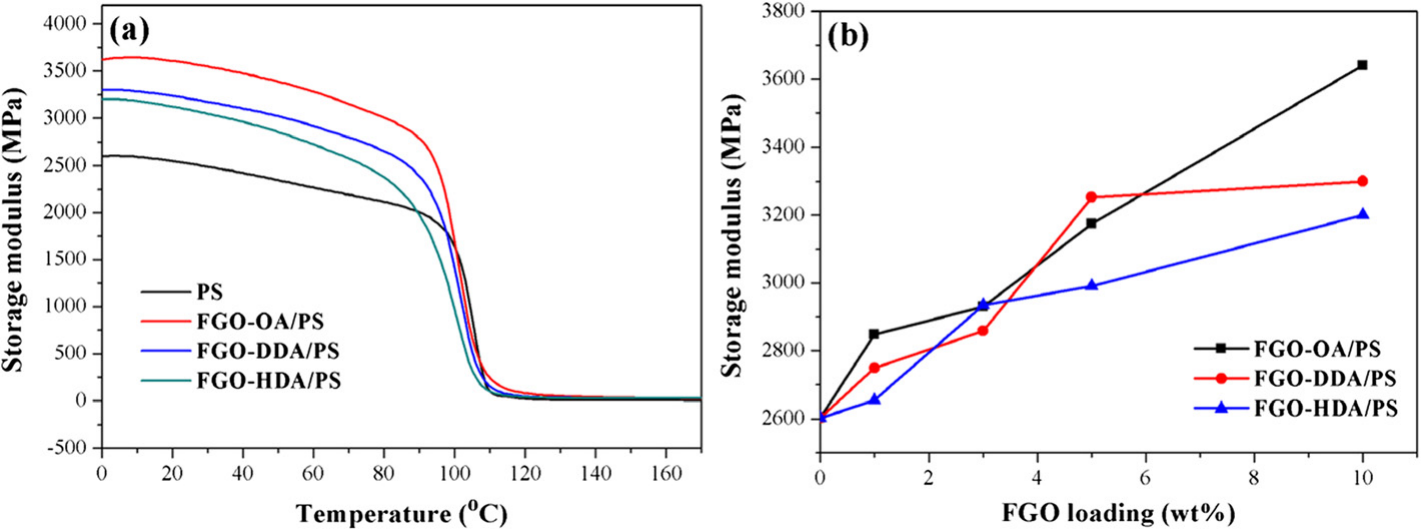
**The storage moduli of the composites. (a)** With a 10 wt.% loading. **(b)** As a function of the FGO loading at 4°C.

The mechanical properties were measured using DMA, as shown in Figure 4a,b. The storage moduli of the pristine PS and FGO/PS composites increased proportionally to the FGO loading (1 to 10 wt.%). The relative increase in the storage modulus was around 40% for FGO-OA/PS corresponding to a FGO-OA loading of 10 wt.% in the glassy region. In our previous study, chemically converted graphene (CCG) without functionalization showed limited dispersion in the PS matrix at a higher graphene loading, resulting in a maximum modulus increase of 28% at 4 wt.% loading of CCG [4]. Contrary to the thermal stability, as the alkyl chain length increased, the modulus decreased. This behavior can be attributed to the crumpled and agglomerated conformation of the FGOs with longer alkyl chains (Figure 2h), which is not an ideal conformation for stretch transfer because these conformations have the tendency to unfold rather than stretch in-plane under an applied tensile stress. A similar result was also observed in the moduli obtained as a function of the FGO content. As shown in Figure 4, FGO-OA, which has shortest alkyl chain length, exhibited the largest modulus increase as a function of the FGO content, which also indicates that the relatively flat morphology of FGO-OA in the PS matrix is more effective against an applied tensile stress.

The glass transition temperatures (*T*_g_) of FGO/PS composite obtained from the tan δ curves are shown in Table 1. Compared with the *T*_g_ of pristine PS (110.4°C), the *T*_g_ values of FGO/PS slightly increased for low FGO loading, up to 3.0 wt.% for FGO-OA/PS and FGO-DDA/PS and only 1.0 wt.% for FGO-HDA/PS, and then decreased for the higher FGO loading. The increase of *T*_g_ at low loading can be attributed to the restricted movement of the PS chains. In the case of FGO-HDA/PS, this tendency was not clear. As described in the above section, the tangled and agglomerated conformation of FGOs with longer alkyl chains of HDA had little effect on the chain movement of the PS chains but acted as a spacer between the PS chains [11,26]. However, as the loading of the FGOs increased, all the *T*_g_ values of FGO/PS decreased. This can be attributed to the increased spaces between the PS chains at the higher FGO loadings, regardless of the chain length of the alkylamines.

**Table 1:** **Glass transition temperatures obtained from the tan** δ **curves**

**FGO loading (wt.%)**	**FGO-OA/PS (°C)**	**FGO-DDA/PS (°C)**	**FGO-HDA/PS (°C)**
0.0	110.44	110.44	110.44
1.0	111.95	111.44	111.44
3.0	112.45	112.43	110.36
5.0	111.19	110.44	110.94
10.0	108.67	109.17	108.42

## Conclusions

Three types of FGO/PS composites were successfully prepared by solution blending. FGOs in the form of grafted alkylamines showed excellent dispersion over PS even at 10 wt.% loading. The dispersed FGOs formed different morphologies over the PS matrix due to the steric effects resulting from the different chain lengths of the alkylamines. All of the FGO/PS composites possessed improved thermal properties and storage moduli with FGO loading. FGO-HDA/PS, which has the longest chain length, showed the best thermal stability compared to other alkylamines. On the other hand, the storage modulus of the FGO-OA/PS composite achieved a maximum value of 3,640 MPa at 10 wt.% FGO-OA loading, which corresponded to 140% of the pristine PS. The functionalization of GO with alkylamines is thought to improve the compatibility of GO with various low-polar polymers due to their good interfacial interaction.

## Competing interests

The authors declare that they have no competing interests.

## Authors’ contributions

The work presented here was performed in collaboration of all authors. JJ designed and performed the work, analyzed the data, and drafted the manuscript. VHP and BR designed and supervised the research work. SHH revised the manuscript. JSC supervised and drafted the manuscript. All authors read and approved the final manuscript.
